# Resting-state functional heterogeneity of the right insula contributes to pain sensitivity

**DOI:** 10.1038/s41598-021-02474-x

**Published:** 2021-11-25

**Authors:** Dániel Veréb, Bálint Kincses, Tamás Spisák, Frederik Schlitt, Nikoletta Szabó, Péter Faragó, Krisztián Kocsis, Bence Bozsik, Eszter Tóth, András Király, Matthias Zunhammer, Tobias Schmidt-Wilcke, Ulrike Bingel, Zsigmond Tamás Kincses

**Affiliations:** 1 Department of Neurology, Faculty of Medicine, University of Szeged, Szeged, Hungary; 2 Institute of Diagnostic and Interventional Radiology and Neuroradiology, University Hospital Essen, Essen, Germany; 3 Neuroimaging Research Group, Department of Radiology, Albert Szent-Györgyi Clinical Center, University of Szeged, Semmelweis u. 6, Szeged, 6725 Hungary; 4 Department of Psychiatry, Faculty of Medicine, University of Szeged, Szeged, Hungary; 5 Department of Neurology, University Hospital Essen, Essen, Germany; 6 Institute of Clinical Neuroscience and Medical Psychology, University of Düsseldorf, Düsseldorf, Germany; 7 Mauritius Therapieklinik, Strümper Str. 111, 40670 Meerbusch, Germany

**Keywords:** Cognitive neuroscience, Perception

## Abstract

Previous studies have described the structure and function of the insular cortex in terms of spatially continuous gradients. Here we assess how spatial features of insular resting state functional organization correspond to individual pain sensitivity. From a previous multicenter study, we included 107 healthy participants, who underwent resting state functional MRI scans, T1-weighted scans and quantitative sensory testing on the left forearm. Thermal and mechanical pain thresholds were determined. Connectopic mapping, a technique using non-linear representations of functional organization was employed to describe functional connectivity gradients in both insulae. Partial coefficients of determination were calculated between trend surface model parameters summarizing spatial features of gradients, modal and modality-independent pain sensitivity. The dominant connectopy captured the previously reported posteroanterior shift in connectivity profiles. Spatial features of dominant connectopies in the right insula explained significant amounts of variance in thermal (R^2^ = 0.076; p < 0.001 and R^2^ = 0.031; *p* < 0.029) and composite pain sensitivity (R^2^ = 0.072; *p* < 0.002). The left insular gradient was not significantly associated with pain thresholds. Our results highlight the functional relevance of gradient-like insular organization in pain processing. Considering individual variations in insular connectopy might contribute to understanding neural mechanisms behind pain and improve objective brain-based characterization of individual pain sensitivity.

## Introduction

The insular cortex is a functionally diverse brain region that is central to several aspects of pain processing, and shapes the subjective experience of the individual when encountering painful stimuli^1,2^. Classically, it is thought to exhibit a dichotomous functional organization. The anterior part is more involved in the cognitive-emotional aspect of pain modulation, whereas the posterior part relates more to nociception, as it is able to encode the modality, intensity and location of a painful stimulus^1,3–5^. The two parts exhibit a heterogeneous cytoarchitecture^6^ and have different connections from the ascending spino-thalamo-cortical pathways as well^7^. Studies suggest a posteroanterior information propagation pattern, likely driven by the convergence of nociceptive inputs in the anterior insula after lower-level processing in the posterior part^3^. This concept is in line with the results of fMRI studies ascribing multisensory integration and higher-order pain modulation to the anterior insula^2^. The anterior–posterior dichotomy view has been challenged many times, and studies seeking an optimal functional partition describe numbers of subregions ranging from two to as many as 13^8^. It seems that characterizing the organization of the insular cortex in terms of discrete subregions does not produce consistent divisions, and recent studies showed that the distribution of both anatomical and functional connections in the insula can be better modeled as a spatially continuous, gradual change in connectivity profiles^9,10^. These models exploit graph-based representations of functional connectivity profiles to construct similarity matrices, from which gradients of functional similarity can be derived using several dimensionality reduction techniques (e.g. the Laplacian Eigenmaps algorithm^11^ or diffusion embedding^12^). The derived gradients can characterize axes of functional organization across the whole brain^13^ or in pre-defined regions of interest; a prominent method employing the latter approach is connectopic mapping, which has also proven useful in extracting multiple overlapping gradients^14^. Gradient-based models have already been employed to relate insular organization to behavior, explaining a range of cognitive, affective and sensorimotor measures previously tied to the insula^10^. While initial evidence based on electrophysiological studies implies that pain-related activation follows a gradual, posteroanterior pattern that is driven by connectivity differences along the posteroanterior axis^3^, the gradient-like functional organization of the insula has not yet been directly linked to pain processing. Such a link could push forward related research in two ways. First, describing insular connectivity in terms of functional gradients might reveal a clearer link between insula function and individual pain sensitivity, since subregions of the insula work together to influence the formation of a painful experience, possibly integrated by connectivity differences along the posteroanterior axis. Although numerous other brain regions are involved in pain processing, the unique anatomical and functional organization of the insula lends itself well to gradient-based models. Second, it would render connectivity gradients as a novel, low dimensional component in predictive modelling approaches aiming at the development of imaging biomarkers of pain^15–17^. Therefore, we hypothesized that a gradient-based characterization of resting state functional organization in the insula is directly associated with individual pain sensitivity. In this study, we investigate whether features of connectivity gradients in the insula explain a significant part of individual pain sensitivity over and above known influencing factors, such as sex, age, and, in the case of women, the day of the menstruation cycle. To assess the possibility of multiple overlapping, meaningful gradients, we employ the connectopic mapping approach. Furthermore, we link our findings to current integrative descriptions of the role the insula plays in pain processing, and discuss how low dimensional representations of functional connectivity in the insula might be integrated into efforts searching for imaging markers of individual pain sensitivity.

## Results

### Quantitative sensory testing

Inclusion and exclusion criteria were the same as in^17^ (see “Methods” section for further details). Pain thresholds for cold (mean +/− standard deviation: 15.87 +/− 8.15 °C), heat (mean +/− standard deviation: 43.44 +/− 3.36 °C) and mechanical stimuli (mean +/− standard deviation: 46.92 +/− 47.92 mN) were within the normative range for all participants as described in^18^. For a detailed description of participant level pain threshold data, see the Supplementary material and https://github.com/spisakt/RPN-signature.

### Connectopic mapping

**Figure 1:**
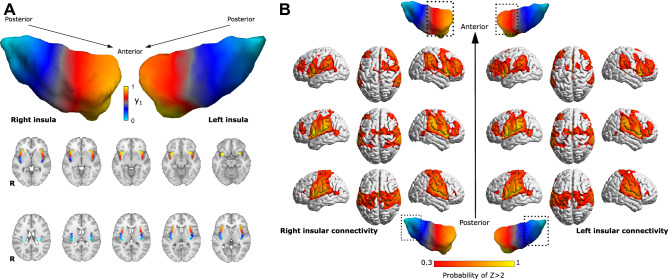
Average connectopy of the insular cortex. (**A**) Average connectopic maps overlaid on the MNI152 template, displayed in neurological orientation. Bilateral insular cortices show a similar posteroanterior trajectory of change in functional connectivity profiles. y_1_: dominant connectopy. (**B**) Heatmaps showing the voxel wise probability of Z-transformed correlation > 2 with the anterior, posterior and middle part of the right insula taken over all participants, overlaid on the ICBM152 template in neurological orientation. Masks for different parts of the insula were derived by dividing the average connectopy into three parts (0 < 0.3 < 0.7 < 1). 3D representations were created using BrainNet Viewer^42^.

The average dominant connectopy across all participants showed a rostrocaudal gradient of connectivity profiles in the insula on both sides. Connectivity changes along the main axis were similar in the bilateral insulae and exhibited the previously reported profiles. The anterior insula mainly showed the strongest connections to dorsolateral prefrontal, anterior cingulate areas and inferior parietal lobules, whereas the posterior part was connected to the parietal operculum and somatosensory areas (see Fig. 1). We observed no significant connectivity differences between the left and right insula.

The second and third connectopies were highly variable and inconsistent across participants, and did not show any association to modal pain thresholds and composite pain sensitivity.

**Figure 2:**
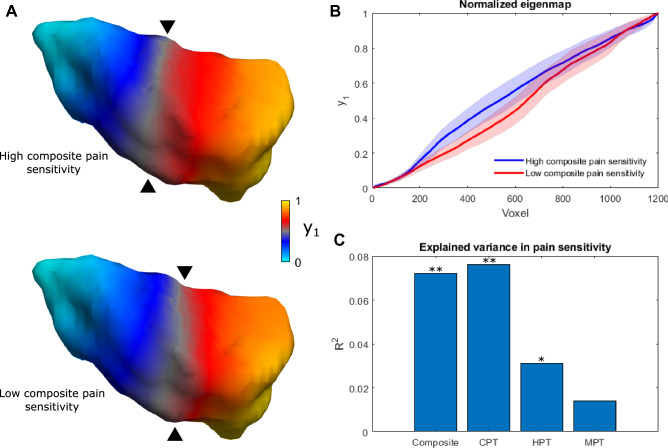
Connectopy of the right insula is associated with pain sensitivity scores. (**A**) The average connectopy of the right insula in participants with high and low composite pain sensitivity (chosen as the upper and lower quintile (< 20% and > 80%) of composite pain sensitivity scores). Apart from posterioanterior steepness, differences in finer spatial variation also contribute to the effect. (**B**) Differences of the connectopy curve in participants with high and low composite pain sensitivity. Shaded areas depict the 95% confidence interval. (**C**) Bar plot showing the contribution of trend surface model parameters to pain sensitivity scores. **p < 0.01, *p < 0.05, y_1_: dominant connectopy, Composite: composite pain sensitivity, CPT: cold pain threshold, HPT: heat pain threshold, MPT: mechanical pain threshold.

The spatial organization of individual dominant connectopies in the right insula (summarized with the TSM parameters) explained a significant amount of variance in the cold and heat pain thresholds (R^2^ = 0.076; *p* < 0.001 and R^2^ = 0.031; *p* < 0.029) and composite scores (R^2^ = 0.072; *p* < 0.002) of participants, apart from nuisance effects (see Fig. 2). Individual coefficients did not correlate with pain sensitivity scores. Regarding the left insula, connectopy features did not explain significant amounts of variance in either pain threshold.

TSM parameters of the dominant connectopy did not differ significantly between males and females after correcting for multiple comparisons.

## Discussion

In this study, we showed that individual spatial patterns of the dominant connectopy in the insula explain a significant amount of variance in both thermal and composite pain sensitivity measured on the contralateral forearm, over and above established biological factors that contribute to pain sensitivity (age, sex and day of the menstrual cycle in women). On a group level, spatial features of the dominant connectopy explained 7.6% and 3.1% variance in modal (cold and heat) pain thresholds and 7.2% variance in modality independent composite scores, which proved to be statistically significant. For comparison, a predictive model of pain thresholds deploying whole brain functional connectivity patterns in our previous study explained 17% and 18% variance during out-of-sample validation analyses^17^. Consequently, the layout of functional connectivity in the insula might provide additional information that might improve current resting-state fMRI-based predictions of subjective pain thresholds.

The dominant connectopy in our sample exhibits a rostrocaudal main axis that falls in line with previous descriptions of anatomical and functional organization in the insula^9,10^. Earlier studies also show that differential connections along the topology of the insula scale in strength with pain-relevant behavioral variables, e.g. pain vigilance and awareness^16^. Accordingly, it is mainly the steepness of the gradient along the rostrocaudal axis that contributes to the variance in our participants’ pain sensitivity scores, as depicted in Fig. 2: larger stepwise connectivity changes in the anterior insula come with lower pain sensitivity scores, whereas larger stepwise changes of connectivity in the posterior insula were associated with higher pain sensitivity. This is consistent with the idea of anterior and posterior dominant individuals in terms of insular connectivity described in^10^. However, single TSM parameters did not explain significant amounts of variance in pain sensitivity scores on their own, suggesting that finer spatial variation is also important on top of a simple posteroanterior connectivity gradient. As depicted in Fig. 1, the main connectopy represents a shift from the typical posterior insular connectivity profile (consistent with reports of stronger connections to parietal somatosensory and cingulate cortices^8^, associated with a role in temperature sensation and the processing of painful thermal stimuli^1,19^) to the typical anterior insular connectivity profile (in line with previous studies reporting mainly connections to dorsolateral prefrontal, anterior cingulate cortices, associated with cognitive and emotional modulation of pain^3,8^). Since previous studies describe a posteroanterior convergence of pain-related information in the insula^3^, the link between spatial features of connectivity change and pain sensitivity could suggest that the dominant connectopy captures (at least in part) the functional organization of this system. In our sample, larger stepwise connectivity changes in the anterior part were associated with lower pain sensitivity. Consistently with this, participants exhibiting a steeper diversity curve in the anterior insula scored higher on measures of positive affect, self-efficacy and emotion recognition in the study by Tian and Zalesky^10^, which, in light of our results, might represent more effective mechanisms of coping with the painful experience that results in lower sensitivity to painful stimuli (e.g. studies show that the anterior insula performs pain-specific integration of pain intensity with expectation^20,21^). Current opinions indicate that a subjective experience of pain arises from the integration of nociceptive, cognitive and emotional information^22^. The anterior insula connects to prefrontal and anterior cingulate cortices, which are implicated in cognitive pain modulation^15,23^, and these connections were shown to be important features in predicting individual pain sensitivity^17^. Additionally, in our recent study we found that morphological and functional network properties of the posterior insula are also associated with pain sensitivity^24^. Based on this, the anterior-dominant connectopy might represent a more effective connectivity layout in terms of top-down pain modulation. Our finding that the modality-independent composite pain sensitivity is also explained by the dominant connectopy supports this further. A possible link between the layout of functional connectivity in the insula and pain sensitivity might be further supported by studies reporting functional and structural reorganization of the insula in chronic pain conditions. A recent fMRI study employing graph theoretical techniques in a fibromyalgia cohort found that hub topology was different in fibromyalgia patients, with insular regions becoming prominent, functionally central hubs scaling with clinical pain and glutamate levels^25^. Another study reported that the altered connectivity of the right anterior insula was central to changes in brain network connectivity in painful knee osteoarthritis patients^26^. In our recent study, we found altered temporal dynamics of connectivity in the right insula of migraine patients that scaled with headache frequency^27^. Further studies are needed to investigate whether these alterations are reflected in the dominant insular connectopy in chronic pain conditions.

Remarkably, in the current study, only the connectopy of the contralateral insula is connected to pain sensitivity, which is consistent with the organization of supraspinal nociceptive pathways and the known right hemispheric dominance in pain-related processing^28,29^. In accordance, previous studies involving direct electrical stimulation of the insular cortex in epilepsy patients reported painful sensations in response to the stimulation, contralateral to the stimulated side^30^. Conversely, electrical potentials elicited by painful laser-based heat stimuli were picked up in the insular cortex both contralateral and ipsilateral to the stimulation site, although evoked potentials in the ipsilateral insular cortex were recorded after a short latency^31^.

Although touch sensation and mechanical pain is also processed in the insula^32^, we did not find a significant association between mechanical pain sensitivity and the dominant connectopy. Prior studies using intracerebral EEG recordings in epilepsy patients reported low-frequency phase-locked local field potentials and gamma-band oscillations (which are considered a correlate of supramodal activity) in the insula on both sides in response to mechano-nociceptive stimulation, however, the amplitude and latency of LFPs and GBOs were different from those elicited by thermal painful stimuli^33^. Additionally, an fMRI study of somatotopic representations for heat and mechanical painful stimuli in the insula found multiple, overlapping representations for these modalities^5^. In light of these reports, it is possible that there are modality-dependent differences in the processing of painful stimuli that are reflected in the functional organization of the insula. However, a lack of association between the dominant connectopy and mechanical pain sensitivity might also be related to the higher noise inherent in the measurement of mechanical pain thresholds.

There are aspects of our study that prompts for further studies. The effects of preprocessing on the estimation of cortical gradients have not yet been fully investigated. Here we employed a preprocessing pipeline similar to that in the original paper, and repeated the analysis with a different, more stringent pipeline which yielded similar results (see Supplementary material).

Since we only measured pain sensitivity unilaterally, more studies are needed to ascertain the laterality of the link between insular functional organization and pain sensitivity. Finally, pain is a complex phenomenon involving many regions of the brain, and here we use only one of them to characterize pain sensitivity. Future studies might examine and relate functional heterogeneity in multiple brain regions to pain sensitivity to get better predictions and complement current imaging markers of individual pain thresholds.

## Conclusions

Characterizing insular connectivity in terms of a low-dimensional representation, such as functional gradients, provides a parsimonious description that can be used to explain both modal and modality-independent contralateral pain sensitivity. This provides a more direct link between insular connectivity and subjective pain sensitivity, and supports earlier accounts of anterior and posterior dominance in terms of insular connectivity. Therefore, functional gradients of the insula might be utilized as complementary markers in objectively predicting individual pain thresholds.

## Methods

### Participants

To investigate the relationship between functional organization in the insula and pain thresholds, we used our dataset published in^17^, which contains data from 116 young healthy participants collected at three centers (University of Szeged, Hungary, Ruhr University Bochum and University Hospital Essen, Germany). From this dataset we included a total of 107 participants (N_Szeged_ = 30, N_Bochum_ = 30, N_Essen_ = 47, mean age: 25.21 +/− 3.54 years, 53 males, 54 females, all right-handed) based on the availability of MRI, QST and demographic data. Any participants with neuropsychiatric or chronic pain conditions (e.g. migraine), those who took medication regularly (except for oral contraceptives), or recently used analgesics were excluded. Participants were asked to refrain from consuming alcohol both before the MRI and QST measurements and on the previous day. Written informed consent was obtained from all participants in accordance with the Declaration of Helsinki, and the local or national ethics committees approved the study (reference numbers 4974-14, 18-8020-BO and 057617/2015/OTIG at Ruhr University Bochum, University Hospital Essen and ETT TUKEB Hungary, respectively). MRI measurements and quantitative sensory testing took place on different dates, with an average difference of 2.4 days.

### Quantitative sensory testing

Individual pain thresholds for cold (CPT), heat (HPT) and mechanical stimuli (MPT) were determined according to the quantitative sensory testing protocol (QST), which is described in detail in^18^. Here we give a brief summary of the procedure. Sensory measurements were carried out on the left palmar forearm. The thresholds were determined using a method of limits, meaning the application of increasing and decreasing temperatures to the skin via a modular sensory analyzer (MSA) thermal stimulator (Somedic, Hörby, Sweden) in the Bochum sample and Pathway thermal stimulators (Medoc Ltd., Ramat Yishai, Israel) in the Szeged and Essen samples. In all cases, advanced thermal stimulator (ATS) thermodes with a baseline temperature of 32 °C were used on a 30 × 30 mm skin surface. Participants indicated the onset of pain by pressing a button. Mechanical pain thresholds were determined using the pinprick test, and were log-transformed for further calculations according to^18^. Modal pain thresholds were then Z-transformed and multiplied by − 1 in the case of heat and mechanical pain thresholds so that higher values denoted higher sensitivity to pain in all three modalities^17^. In addition to modal pain thresholds, a modality-independent composite pain sensitivity score was also calculated as the arithmetic mean of the three Z-transformed (and in the case of heat and mechanical thresholds, inverted) modal pain thresholds according to^17^ and^34^ for all participants.

### Image acquisition

We refer to Table 4 in^17^ for the full list of acquisition parameters in the three centers. Briefly, for all participants, a T1-weighted structural image (MPRAGE sequence in Essen and Bochum, 3D-FSPGR in Szeged, 1 mm^3^ spatial resolution), and a T2*-weighted functional scan (BOLD EPI sequence, TR = 2500 ms in Szeged and Bochum, TR = 2520 ms in Essen, voxel size 3 × 3 × 3 mm^3^ in Szeged and Bochum, 2.45 × 2.45 × 3 mm^3^ in Essen) were acquired. The number of functional scans was 240, 200 and 290 for Szeged, Bochum and Essen, respectively, resulting in scan times of 8 min 37 s, 12 min 11 s and 10 min. The scanners used were a GE Discovery MR750W 3T (Szeged), a Philips Achieva X3T (Bochum) and a Siemens Magnetom Skyra 3T (Essen). During the acquisition, participants were instructed to lie motionless while keeping their eyes open.

### fMRI preprocessing

We used the FMRIB Software Library to preprocess the data (FSL v5.0.10^35^). The first 5 volumes were discarded in order to avoid saturation effects. Preprocessing steps included motion correction via MCFLIRT, brain extraction, slice-timing correction, intensity normalization, two-stage registration to 2 mm MNI space using a boundary-based registration algorithm and FNIRT, nuisance regression with the six estimated motion parameters and the white matter and cerebrospinal fluid time courses, high pass filtering with a cutoff at 0.01 Hz and removal of linear trends. Before calculating the connectopies, we normalized the data to zero mean and unit standard deviation.

### ROI definition

To acquire an accurate delineation of the bilateral insulae, we ran Freesurfer’s recon-all^36^ on the standard 2 mm MNI template, and used the resulting parcellated cortical surface to define the region of interest on both sides.

### Connectopic mapping and trend surface modelling

Connectopic mapping is a novel data-driven approach that was demonstrated to reproducibly trace gradients of functional organization in several brain areas and studies used it to link spatial patterns of these gradients to cognitive and behavioral measures^14,37,38^. Although the details are described elsewhere^14^, we proceed with a brief description. In a nutshell, the analysis begins by computing the functional connectivity of each voxel inside the region of interest to spatial modes in a target mask (in our case, this is the entire brain), derived using singular value decomposition. Functional connectivity is characterized using the Pearson correlation coefficient. This way, a functional connectivity fingerprint is obtained for each voxel in the region of interest. Then the similarity of the these fingerprints is characterized using the eta-squared similarity measure^39^, which results in a similarity matrix. Using the Laplacian eigenmaps algorithm, a non-linear dimension reduction technique^11^, the similarity matrix is then transformed into a connected graph and decomposed. The resulting eigenvectors of the graph Laplacian represent modes of connectopic organization, defining gradients in functional organization termed connectopies. These connectopies are orthogonal to each other, therefore the procedure is able to distinguish between overlapping gradients in functional organization, as demonstrated e.g. in the visual cortex^14^.

**Figure 3:**
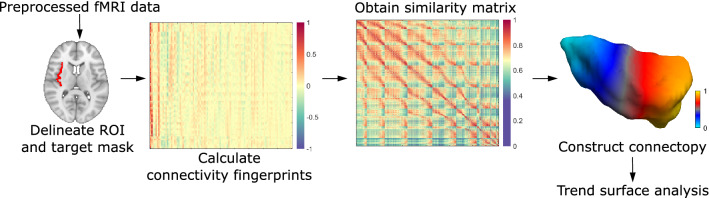
A schematic representation of the connectopic mapping approach. For further details, see the Methods section.

In order to perform statistics on the connectopies, the approach uses a trend surface model to describe the spatial pattern of the gradient. This model is essentially a spatial regression model, where, in the simplest (first order) case, a voxel´s value is predicted by the linear combination of its coordinates^40^. We used a third order model as a trade-off between explained variance and model parsimony, since higher order models explained minimal amounts of additional variance in the average gradient’s spatial pattern and previous studies involving large databases also employed a third order model in several different brain structures^14,37,38^. In this case, the model includes 9 parameters altogether, corresponding to the three axes of MNI-space and the second and third power. We fit a trend surface model (TSM) separately for each individual and each hemisphere using Bayesian linear regression, and performed connectopic mapping and trend surface model fitting using the freely available CONGRADS toolbox (https://github.com/koenhaak/congrads). A schematic representation of the connectopic mapping analysis can be seen in Fig. 3.

To investigate whether functional modes apart from the anteroposterior trajectory exist that are relevant to pain thresholds, we extracted the first 3 gradients. Furthermore, in order to visualize connectivity differences that drive connectopies, we divided the dominant connectopy into three parts and calculated whole-brain connectivity from each subdivision. Individual connectivity maps for the three subdivisions were thresholded at Z = 2, binarized and a group-level probability map was calculated for all three subdivisions.

### Statistical analysis

To determine whether the spatial features of connectopies explain a significant amount of variance in pain thresholds, we employed a general linear model-based approach. In particular, we tested whether the participant-level TSM parameters of bilateral insular connectopies explain a significant amount of variance over and above the nuisance variables (age, sex and day of menstruation cycle). For this, we calculated the partial coefficient of determination (R^2^), and assessed its significance via a permutation approach implemented in FSL PALM using 10,000 permutations^41^. To see if there are any sex differences in insular connectopies, we compared them using Mann–Whitney U-tests while correcting for multiple comparisons according to Bonferroni.

## Supplementary Information

Supplementary Information.

## Data Availability

Raw imaging data is available at openneuro.org (ds001900). QST assessments and demographic data are available at https://github.com/spisakt/RPN-signature.
