# Supplementary material for: Resting-state functional heterogeneity of the right insula contributes to pain sensitivity
Source: Sci Rep. 2021 Nov 25;11:22945. doi: 10.1038/s41598-021-02474-x (PMC8617295; doi:10.1038/s41598-021-02474-x)
Supplement: Supplementary file 1 — Supplementary Information. [file 41598_2021_2474_MOESM1_ESM.pdf]

Resting-state functional heterogeneity of the right insula contributes to pain sensitivity

Dániel Veréb, PhD, Bálint Kincses, PhD, Tamás Spisák, PhD, Frederik Schlitt, Nikoletta Szabó, PhD, Péter Faragó, PhD, Krisztián Kocsis, MSc, Bence Bozsik, MD, Eszter Tóth, PhD, András Király, PhD, Matthias Zunhammer, PhD, Tobias Schmidt-Wilcke, PhD Ulrike Bingel, PhD, Zsigmond Tamás Kincses, PhD

Supplementary material

The PUMI pipeline

In order to check whether a different preprocessing pipeline impacts the gradient estimation and the link between insular gradients and pain sensitivity, we repeated the analysis using a more stringent pipeline, the open-source Python software library Pipelines Utilising a Modular Inventory (PUMI, https://github.com/spisakt/PUMI), which is described in detail in [6]. Briefly, the pipeline includes brain extraction and tissue segmentation using FSL BET and FAST for both the structural and functional images followed by the linear and non-linear registration of the structural image to 2mm MNI space using ANTs [1]. The functional image is then corrected for motion using MCFLIRT and outliers are removed using AFNI despike [3]. Nuisance regression entails the use of CompCor [2], plus removing the Friston-24 motion parameters [4] and linear trends. The residual data is smoothed using a 6mm FWHM Gaussian filter and bandpass filtered retaining the 0.008-0.08 Hz frequency range with AFNI's 3DBandpass. Finally, frames exceeding a framewise displacement of 0.15 are scrubbed [5]. The functional image is registered to the structural image using a boundary-based registration algorithm implemented in FSL, then further transformed to 2mm MNI space using the structural-to-standard transformation warp fields.

Results with the PUMI pipeline

The same rostrocaudal gradient appeared in the majority of participants as with the previous pipeline, but less consistently (93 participants out of 107). In participants who exhibited a gradient consistent with the rostrocaudal organization, the TSM parameters explained a significant amount of variance in individual cold thresholds and composite scores ($R^2 = 0.042$; $p < 0.018$ and $R^2 = 0.038$; $p < 0.026$). Similarly to the pipeline described in the main manuscript, we found no link between connectopic organization in the left insula and pain thresholds.

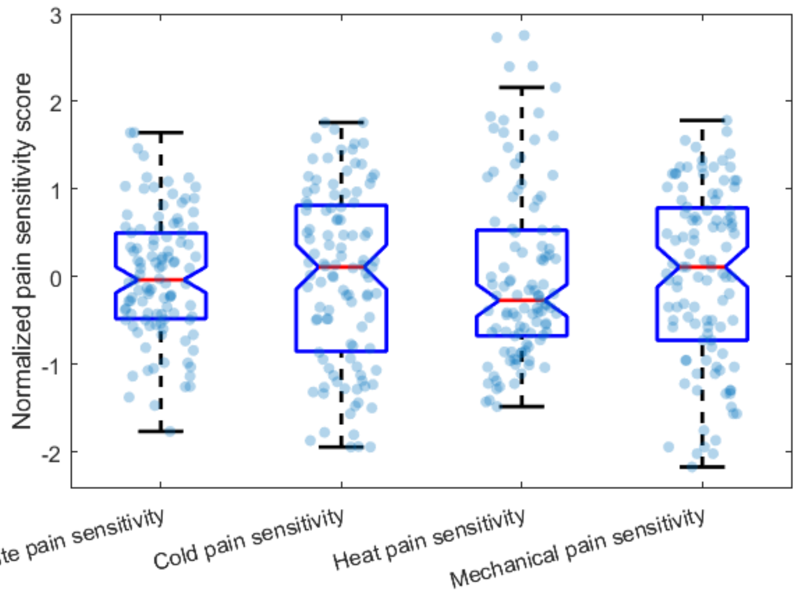

Figure S1.: Distribution of pain sensitivity scores in the study cohort. Boxplots represent the median, interquartile range and 10/90 percentiles. Individual data points are depicted in blue.

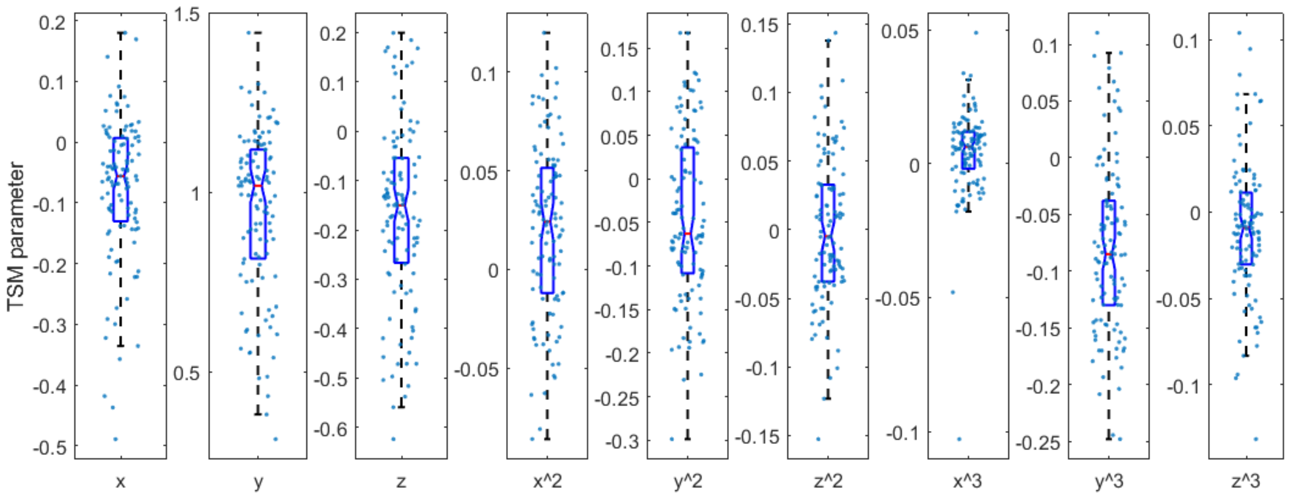

Figure S2.: Distribution of trend surface model parameters for the estimated individual connectopies. Boxplots represent the median, interquartile range and 10/90 percentiles. Individual data points are depicted in blue.

Supplementary references

[1] Avants BB, Tustison NJ, Song G, Cook PA, Klein A, Gee JC. A reproducible evaluation of ANTs similarity metric performance in brain image registration. NeuroImage 2011;54(3):2033-2044.

[2] Behzadi Y, Restom K, Liau J, Liu TT. A component based noise correction method (CompCor) for BOLD and perfusion based fMRI. NeuroImage 2007;37(1):90-101.

[3] Cox RW. AFNI: software for analysis and visualization of functional magnetic resonance neuroimages. Computers and biomedical research, an international journal 1996;29(3):162-173.

[4] Friston KJ, Williams S, Howard R, Frackowiak RS, Turner R. Movement-related effects in fMRI time-series. Magnetic resonance in medicine 1996;35(3):346-355.

[5] Satterthwaite TD, Elliott MA, Gerraty RT, Ruparel K, Loughead J, Calkins ME, Eickhoff SB, Hakonarson H, Gur RC, Gur RE, Wolf DH. An improved framework for confound regression and filtering for control of motion artifact in the preprocessing of resting-state functional connectivity data. NeuroImage 2013;64:240-256.

[6] Spisak T, Kincses B, Schlitt F, Zunhammer M, Schmidt-Wilcke T, Kincses ZT, Bingel U. Pain-free resting-state functional brain connectivity predicts individual pain sensitivity. Nature Communications 2020;11(1):187.
